# Is increased antidepressant exposure a contributory factor to the obesity pandemic?

**DOI:** 10.1038/tp.2016.25

**Published:** 2016-03-15

**Authors:** S H Lee, G Paz-Filho, C Mastronardi, J Licinio, M-L Wong

**Affiliations:** 1 Department of Genome Sciences, John Curtin School of Medical Research, Australian National University, Canberra, ACT, Australia; 2 Pharmacogenomics Research Program, Mind and Brain Theme, South Australian Health and Medical Research Institute and Department of Psychiatry, School of Medicine, Flinders University, Adelaide, SA, Australia

## Abstract

Major depressive disorder (MDD) and obesity are both common heterogeneous disorders with complex aetiology, with a major impact on public health. Antidepressant prescribing has risen nearly 400% since 1988, according to data from the Centers for Disease Control and Prevention (CDC). In parallel, adult obesity rates have doubled since 1980, from 15 to 30 percent, while childhood obesity rates have more than tripled. Rising obesity rates have significant health consequences, contributing to increased rates of more than thirty serious diseases. Despite the concomitant rise of antidepressant use and of the obesity rates in Western societies, the association between the two, as well as the mechanisms underlying antidepressant-induced weight gain, remain under explored. In this review, we highlight the complex relationship between antidepressant use, MDD and weight gain. Clinical findings have suggested that obesity may increase the risk of developing MDD, and *vice versa*. Hypothalamic–pituitary–adrenal (HPA) axis activation occurs in the state of stress; concurrently, the HPA axis is also dysregulated in obesity and metabolic syndrome, making it the most well-understood shared common pathophysiological pathway with MDD. Numerous studies have investigated the effects of different classes of antidepressants on body weight. Previous clinical studies suggest that the tricyclics amitriptyline, nortriptyline and imipramine, and the serotonin norepinephrine reuptake inhibitor mirtazapine are associated with weight gain. Despite the fact that selective serotonin reuptake inhibitor (SSRI) use has been associated with weight loss during acute treatment, a number of studies have shown that SSRIs may be associated with long-term risk of weight gain; however, because of high variability and multiple confounds in clinical studies, the long-term effect of SSRI treatment and SSRI exposure on body weight remains unclear. A recently developed animal paradigm shows that the combination of stress and antidepressants followed by long-term high-fat diet results, long after discontinuation of antidepressant treatment, in markedly increased weight, in excess of what is caused by high-fat diet alone. On the basis of existing epidemiological, clinical and preclinical data, we have generated the testable hypothesis that escalating use of antidepressants, resulting in high rates of antidepressant exposure, might be a contributory factor to the obesity epidemic.

## Introduction

Major depressive disorder (MDD) and obesity are both common, heterogeneous disorders with complex aetiology, and pronounced public health impact.^1, 2^ According to data from the World Health Organization (WHO), MDD has become the second most prevalent cause of illness-induced disability, affecting 350 million people worldwide.^3^ In the United States, costs related to MDD such as medical expenses are estimated to be $210.5 billion a year.^4, 5^ With a lifetime prevalence of 16.2%, MDD is twice as common in women. Moreover, two-thirds of suicides are associated with MDD.^1^ In Australia, according to the 2011–2012 National Survey of Mental Health and Wellbeing, 9.7% of people aged 16–85 years were affected by mood disorders.^6^ In Europe, 1 out of 15 people are known to suffer from MDD.^7^

Concomitantly, obesity is a debilitating epidemic affecting 34.9% of US adults (78.6 million individuals), resulting in estimated annual medical costs of $147 billion in 2008.^8^ Since 1962, the prevalence of obesity among adults more than doubled, from 13.4 to 35.7% among American adults aged 20 and older,^9^ in a manner that parallels the increase in MDD treatment. Besides type 2 diabetes mellitus and cardiovascular diseases (CVDs), obesity has other disabling consequences, including cancer,^10^ sleep disorders^11^ and psychological distress.^12^ In the past few decades, studies have addressed the relationship between obesity and MDD.^13, 14, 15, 16, 17, 18^ Previous clinical findings have suggested that obesity could increase the risk of developing MDD, and *vice versa*.^19, 20, 21, 22, 23, 24^ It has been suggested that obesity and MDD share common pathophysiology to a certain extent, yet the precise pathways and mechanisms for a causal association between MDD and obesity remain unknown.

In United States of America, over the past two decades, the rate of antidepressant use has increased nearly 400%, where antidepressant drugs were the third most prescribed class of drugs for persons aged 18-44 years in 2005–2008.^25^ In 2010, it was reported that 52.3 daily doses were prescribed for every 1000 inhabitants in European countries.^26^ In Australia, 34 million prescriptions were issued for mental health-related diseases in 2013–2014, and 67% (23 million) of those were for antidepressants.^27^ In 2012–2013, mental health-related prescriptions cost the Australian Government over $788 million.^27^ With a rapid rise of individuals taking antidepressants, numerous studies have investigated the effects of different classes of antidepressants on body weight. Despite the concomitant occurrence of the frequent use of antidepressants and the high incidence of obesity in Western societies, the pathways and mechanisms by which antidepressants can induced weight gain remain unclear.

In this review, we examine the pathophysiology of MDD and obesity, and their complex interactions. More specifically, we discuss the role of antidepressant use in weight gain, and the role of interactions with environmental factors, such as stress.

## MDD

The diagnosis of MDD is made in the presence of five or more of the following symptoms for a continuous 2-week period or longer, as described in the Diagnostic and Statistical Manual of Mental Disorders (DSM-5): (i) depressed mood most of the day, nearly every day; (ii) significant weight loss/gain or decrease/increase in appetite; (iii) markedly diminished interest or pleasure in almost all activities; (iv) insomnia or hypersomnia; (v) fatigue or loss of energy; (vi) psychomotor retardation or agitation; (vii) feelings of worthlessness; (viii) diminished ability to concentrate or make decisions; and (ix) recurrent thoughts of death.^28^ MDD is a complex disorder; its exact pathophysiology remains elusive, as does the mechanism of action of antidepressants. Over many decades, the pathophysiology of MDD was thought to be based on the hypothesis of monoamine depletion, supported by the fact that the monoamine oxidase inhibitors (MAOIs), a class of antidepressants, restored physiological levels of monoamine in the brain.^29^ Although monoamine levels can be restored acutely, within a few hours after treatment, full antidepressant action is achieved only after 3–4 weeks of treatment initiation. This gap between immediate biochemical effects and delayed clinical response fostered the development of new theories for the pathophysiological mechanism of MDD that go beyond the monoamine hypothesis.

Other theories for the underlying biology of MDD include the neuroendocrine, neuroimmune and neurotropic hypotheses.^29, 30^ The neuroendocrine theory proposes that the pathophysiological mechanism of MDD involves abnormal homeostasis of the hypothalamus–pituitary–adrenal (HPA) axis, with overactivation of the stress response.^30, 31^ Cortisol levels are often increased in the plasma of MDD patients,^30, 32^ and antidepressant treatment downregulates the HPA axis response.^30^ Moreover, recent evidence points towards the importance of insulin-like growth factor 1 in MDD.^33, 34, 35, 36^ It has been suggested that, in mice, insulin-like growth factor 1 deficiency is accompanied by impaired adult hippocampal neurogenesis and decreased total number of neurons in the hippocampus.^33^ In a mouse model, administration of insulin-like growth factor 1 via intracerebroventricular injection has an antidepressant-like action in rodents.^33, 34, 35, 36^ Furthermore, it has been observed that the adipose tissue-derived hormone leptin has a potential role in MDD: circulating leptin levels are decreased in MDD patients and in individuals who attempt suicide, in comparison with healthy controls.^37, 38, 39, 40^ In contrast, the relationship between antidepressant treatment and leptin levels remains less clear. Schilling *et al.* have shown that amitriptyline or mirtazapine antidepressant treatment increased plasma leptin concentrations, whereas the plasma leptin level remained unaltered with paroxetine and venlafaxine treatments.^41^ Furthermore, intrahippocampal, but not intrahypothalamic administration of leptin led to antidepressant-like action in rodents, suggesting that leptin-induced antidepressant actions were not secondary to leptin-induced metabolic effects.^42^

The neuroimmune theory proposes that immune mediators such as cytokines (e.g., interferons and interleukins) may have a role in MDD.^30^ As those immune mediators modulate key functions such as sleep, appetite, cognition and temperature regulation, any alterations in those mediators can contribute indirectly to the pathogenesis of MDD, by disrupting vital functions.^30^ That theory is substantiated by the observation that the innate immune system is altered in MDD towards a pro-inflammatory state, and by the fact that some antidepressants act by reducing inflammation via cyclooxygenase inhibition.

In addition, cytokines can contribute to HPA axis hyperactivity, and affect the serotonergic, dopaminergic, glutamatergic and monoamonergic systems, contributing to MDD.^43^ Accordingly, pro-inflammatory cytokines stimulate glucocorticoid release by acting at all three levels of the HPA axis: at the paraventricular nucleus level, they stimulate the release of corticotropin-releasing hormone (CRH) level; at the pituitary level, they stimulate the release of adrenocorticotropin; and at the adrenal glands, they stimulate the release of glucocorticoids.

The neurotropic theory is a newer hypothesis for MDD, and it proposes that antidepressant treatment leads to acute restoration of monoamine levels in the brain, followed by changes in neuroplasticity via increased synaptic contacts and dendritic arborisation.^44^ The neurotropic hypothesis implies that neurotropic factors are the key factors in antidepressant action.^29^ These include the nerve growth factor (NGF) and the neurokine or neuropoetin superfamilies. The NGF superfamily includes NGF, brain-derived neurotropic factor (BDNF), neurotrophin-3 and neurotrophin-6. The antidepressant effect via BDNF has important roles in supporting neuronal survival and maintaining neuroplasticity.^45^ In rodent models of stress-induced depression, lower levels of BDNF in the hippocampus were restored by antidepressant treatment.^44, 46^ Furthermore, antidepressant treatments have failed to elevate hippocampal neurogenesis at the subgranular zone in a mouse model of absence of BDNF–tyrosine receptor kinase B (BDNF-TrkB) signalling.^47^ Consequently, neurotropic factors, such as BDNF, may have an important role in the mechanism of antidepressant action.

## Obesity

Obesity is primarily defined as the excess of fat mass of sufficient magnitude to produce adverse health consequences. It is diagnosed based on total body weight in relation to height (that is, body mass index (BMI) of 30 kg/m^2^ and over), whereas overweight is diagnosed in the presence of BMI between 25 and 29.9 kg/m^2^.^48^ As those diagnostic criteria do not take into account fat content, other measures of adiposity can also be used for the proper assessment of metabolic risk, such as waist circumference, waist-to-hip ratio, total body fat and body fat percentage. By employing those measures, one can identify individuals with normal body weight and excessive fat content ('metabolically obese, normal weight'), who are also at risk for metabolic diseases. The overly reductionistic explanation of the pathophysiology of obesity as solely due to energy imbalance resulting from excessive food intake and insufficient energy expenditure has evolved over time to become more nuanced. It is now known that the causes of obesity are complex, and many factors including environment, genetics, culture, food choices and hormonal factors contribute to obesity.^49^

Obesity is one component of a cluster of risk factors that increases the risk for the development of type 2 diabetes mellitus and CVD, known as metabolic syndrome (MetS).^50^ There is no consensus regarding the diagnosis of MetS; however, in 2009, a joint statement by various medical organisations was published, stating that obesity and insulin resistance are not prerequisites for MetS; three of the five following components would suffice for a diagnosis of MetS: elevated waist circumference (a measure of visceral obesity), high blood pressure (or antihypertensive drug treatment in a patient with a history of hypertension), high fasting hyperglycaemia (or drug treatment of elevated glucose), elevated triglycerides (or drug treatment for elevated triglycerides) and reduced high-density lipoprotein (HDL)-cholesterol (or drug treatment for reduced HDL-cholesterol).^51^

## MDD and obesity

Obesity and MDD have in common several biological aspects, as described by Bornstein *et al.*^52^ Both disorders frequently co-exist, causing substantial health problems. However, those disorders sometimes do not occur concurrently; obesity can follow depression that occurred earlier in life. On the one hand, depressed mood can be a side effect of obesity treatments, which is further confounded by changes in lifestyle. On the other hand, weight gain can be a side effect of antidepressant treatments. From the pathophysiological point of view, the regulation of mood and body weight is characterised by shared neuropeptidergic and neurotransmitter systems, such as CRH, neuropeptide Y (NPY), serotonin and norepinephrine. Both disorders can lead to CVD, and genetic polymorphisms may underlie the predisposition both to CVD and to depression. Finally, most drugs used in depression and in obesity predominantly affect either serotonin or norepinephrine.

In the past few decades, studies have addressed the relationship between obesity and MDD, and they have suggested that both disorders share a common pathophysiology to a certain extent. However, because of the heterogeneity of clinical data and the methodology used in different studies, variations do exist across studies. Cross-sectional and longitudinal studies have been conducted in order to understand the casual relationship between MDD and obesity, and *vice versa*.^13, 14, 15, 16, 17, 18^ Several studies have pinpointed that obesity is positively associated with MDD. In the cross-sectional study conducted by Rosmond *et al.*, waist circumference (a measure of visceral adiposity) was associated with symptoms of MDD among Swedish men.^13^ In another study, obesity was associated with increased risk for affective disorders in a cluster sample of young German women.^15^ In the third National Health and Nutrition Examination Survey (NHANES-III), obese individuals with BMI>40 were more likely to suffer from MDD than individuals with BMI of 30–34.9  kg/m^2^.^16^ In longitudinal studies with a 5-year follow-up, obese individuals had an increased odds ratio of 2.13 for MDD, indicating that obese individuals are twice as likely to suffer from MDD in comparison with non-obese individuals.^17^ Another study suggested that obesity is associated with an ~25% increase in odds of mood and anxiety disorders.^53^ In a meta-analysis of cross-sectional studies in the general population, a significant positive association between depression and obesity in the general population was observed, which was stronger among women.^54^ Another meta-analysis evaluating the longitudinal, bidirectional evidence of the association between depression and obesity showed that obesity increased the risk of onset of depression (unadjusted odds ratio (OR), 1.55), and that depression also increased the odds for developing obesity (OR, 1.58).^55^

MDD may in some cases be the causal trigger leading to obesity. This hypothesis is supported by the meta-analysis by Luppino *et al.*, where obesity at baseline increased the risk of onset of depression at follow-up (unadjusted OR of 1.55).^56^ In a New Zealand-based longitudinal study, adolescent girls suffering from MDD had a twofold increase in the risk for developing obesity in adulthood than non-depressed ones.^18^ Other studies have also demonstrated that depressive symptoms during childhood are associated with weight gain and obesity during young adulthood,^57^ and that baseline depression is associated with a 5-year increase in visceral obesity.^58^

In addition to these clinical findings, obesity and MDD are influenced by gene–environment interactions.^59^ In fact, there have been association studies of obesity that identified genes that are hypothetically involved in depression. Two of them, the glucocorticoid receptor *NR3C1* (ref. 60) and the *CRH* genes^61^ are related to the dysregulation of the HPA. Similarly, the *BDNF* Val66Met polymorphism is also associated with obesity and depression.^62^

## MDD and MetS

Studies have shown that the prevalence of MetS is greater in individuals with MDD symptoms.^19, 20, 21, 22^ Women with a history of MDD have twice the chance of developing MetS than women without such history.^20^ In longitudinal studies of 7–15 years of follow-up, women with symptoms of MDD and increased tension and anger at baseline had a higher risk of developing MetS.^24^ Furthermore, the risk for MetS was elevated from 1.21- to 2.12-fold (95% confidence interval (CI) 1.00–4.25) in individuals with more severe MDD symptoms or very stressful life events.^23^ These cross-sectional and longitudinal studies have shown that MDD symptoms and stressful life events increase the risk of developing MetS. Thus, current studies clearly support that there is a relationship between MDD and MetS, which also appears to be bidirectional: according to a recent meta-analysis of cross-sectional and cohort studies, the pooled crude OR between depression and MetS was 1.42 (95% CI 1.28–1.57). In the same meta-analysis, the pooled adjusted OR was 1.49 (95% CI 1.19–1.89) for MetS predicting depression risk, and 1.52 (95% CI 1.20–1.91) for depression predicting MetS risk.^63^ Other meta-analyses showed that patients with MDD had a higher MetS prevalence (OR 1.54 (95% CI 1.21–1.97)), with higher risk for hyperglycaemia (OR 1.33 (95% CI 1.03–1.73)) and hypertriglyceridaemia (OR 1.17 (95% CI 1.04–1.30)).^64^ A common pathophysiological pathway between MDD and metabolic dysregulation involves the HPA axis, as described below.

## The interplay between stress, MDD and obesity

Chronic stress is a state where there is persistent activation of the HPA axis.^65^ Moreover, the HPA axis is also dysregulated in obesity, making this axis a shared pathophysiological pathway with MDD.^65^ During stress, CRH is released from the paraventricular nucleus of the hypothalamus, stimulating the secretion of adrenocorticotropin, which in turn stimulates cortisol secretion from the adrenal glands.^65^ In order to compensate for the resulting hypercortisolism, the negative feedback mechanism of glucocorticoid receptors is activated and downregulates the cortisol stress response.^65^ However, in MDD, an excess amount of cortisol in circulation and the over-expression of mineralocorticoid and glucocorticoid receptor results in decreased negative feedback, leading to enduring hypercortisolism.^66^ Cortisol facilitates lipid accumulation by activation of glucocorticoid receptor; as these receptors are highly expressed in the intra-abdominal and visceral areas, this is one of the major pathways leading to visceral and central obesity, and consequently, metabolic complications.^65^ Inflammation is another pathway that links obesity to MDD,^56^ as both disorders are associated with the activation of inflammatory pathways.^56^ However, studies have also demonstrated a pro-inflammatory state in lean depressed individuals, suggesting that the pro-inflammatory state in MDD may not be primarily related to obesity.^67^ The simultaneous activation of inflammatory pathways by depression and by obesity can only be detrimental. Furthermore, increased insulin resistance, frequently observed in obesity and in pro-inflammatory states, may induce alterations in the brain and increase the risk of MDD.^56^

## Antidepressants

In 1950s, the antidepressant effects of MAOIs were first discovered when tuberculosis patients were treated with iproniazid.^68, 69, 70^ Shortly after iproniazid treatment initiation, patients experienced an antidepressant-like effect with elevated mood. Subsequently, it was shown that iproniazid is a MAOI, with the ability to inhibit the enzyme monoamine oxidase to catabolise monoamines, preventing monoamine depletion in the brain.^69^ Since then, it has been hypothesised that a depletion in other neurotransmitters such as norepinephrine, dopamine and serotonin contributes to cause MDD.^69^ There are different classes of antidepressant drugs, and, primarily, they have been known to work by modulating neurotransmitter levels in the brain.^4^ Older generation antidepressants include MAOIs and tricyclics (TCAs),^4^ whereas newer types of antidepressants have greater specificity and include selective reuptake inhibitors (selective serotonin reuptake inhibitor, SSRI), serotonin and norepinephrine reuptake inhibitors (SNRIs), and norepinephrine and dopamine reuptake inhibitors.^4^ Other antidepressants include tetracyclics and serotonin antagonist and reuptake inhibitors.

Currently, non-selective MAOIs on the market include phenelzine (Nardil), isocarboxazid (Marplan) and tranylcypromine (Parnate).^68^ Selective MAOIs include selegiline (Emsam) and clorgyline.^68^ However, the use of such drugs declined because of their serious side effects, such as those related to food and drug interactions. MAOIs are used as a third or fourth line of the therapy when other types of therapy have failed.^68^

The therapeutic action of TCAs is known to occur via inhibition of serotonin and norepinephrine reuptake.^71^ These include imipramine (Tofranil), amitriptyline (Elavil), nortiptyline (Allegron), trimipramine (Surmontil), protriptyline (Concordin) and iprindole (Prondol).^72^ However, TCAs have multiple nonspecific actions that are associated with side effects.^71^ These nonspecific actions include anticholinergic–antimuscarinic (M1), α-1 adrenergic antagonistic and antihistaminergic activities (H1).^71^ Furthermore, large doses of TCAs inhibit sodium channels, leading to lethal cardiac arrhythmias and seizures.^71^ For this reason, TCA antidepressants are used with caution in patients with high risk of CVD.^73, 74, 75^ Moreover, TCA-induced weight gain is one of the main reasons for the discontinuation of treatment within 1 month.^76^

Since the US Food and Drug Administration's approval of fluoxetine (Prozac) in 1988, SSRIs have been the most prescribed antidepressants on the market.^77^ They act by inhibiting serotonin (5-hydroxytryptamine, 5HT) uptake. Since then, other SSRIs have emerged in the market, and these include citalopram (Celexa), fluvoxamine (Floxyfral), paroxetine (Aropax) and sertraline (Zoloft).^78^ Although SSRIs have been well tolerated by patients because of fewer side effects than previous drug, there is still a substantial profile of adverse drug reactions, such as sexual dysfunction, drowsiness, weight gain, dry mouth, insomnia, fatigue, nausea, dizziness and tremors.^79, 80^ The tolerability of a drug can be assessed by discontinuation rates in clinical trials. The discontinuation rate within 2 months for fluvoxamine was ~70%, followed by fluoxetine (45%) and sertraline (40%).^81, 82, 83^

After the successful development of SSRIs, it was proposed that the simultaneous action of selective noradrenaline and serotonin reuptake could provide better efficacy.^84, 85, 86^ Thus, antidepressant SNRIs are as effective as TCAs, but side effects such as cardiac arrhythmias and seizures are significantly reduced.^84, 85, 86^ SNRIs available on the market include duloxetine (Cymbalta), venlafaxine (Effexor), desvenlafaxine (Pristiq) and milnacipran (Savella).

## Clinical findings on the effects of antidepressant use on body weight

### TCA

In the study conducted by Berken *et al.*, low to moderate doses of the TCAs amitriptyline (150 mg per day), nortriptyline (50 mg per day) and imipramine (80 mg per day) were associated with a mean weight gain of 1.3–2.9 lbs per month, and the trend of weight gain remained linear overtime.^76^ Furthermore, it was suggested that the main reason for patients discontinuing treatment was excessive weight gain.^76^ In more recent studies, TCA antidepressants have been shown to increase the OR for MetS to 2.30 (95% CI 1.21–4.36), and this was independent of depression severity.^87^ In a 4-year follow-up study, the mean weight gain in patients treated with unspecific antidepressants was 4.3%, whereas in control subjects it was 2.5%. Moreover, weight gain for the TCA antidepressant users was 4.7% (Table 1).^88^

Sussman *et al.* observed that during acute treatment with imipramine, 4.9% patients significantly gained weight; this rate increased to 24.5% in long-term treatment.^93^ Nortriptyline (TCA) was associated with an increase in BMI of 0.44  kg/m^2^ over a 12-week period.^94^ Furthermore, 6 months of nortriptyline treatment was associated with an increase of 0.64 kg/m^2^ in BMI.^94^

## SSRIs and SNRIs

Initially, when patients were treated acutely with SSRIs weight loss was observed.^95^ Currently, SSRIs are useful for the treatment of obese patients with binge eating disorder, given their anti-impulsive action. However, their clinical relevance for weight loss in these patients remains unclear. In the recently published Endocrine Society Clinical Practice Guideline on the Pharmacological Management of Obesity, the use of SSRIs is not listed as a therapeutic tool.^96^ Despite the fact that SSRI use has been associated with weight loss when acutely treated, a number of studies have shown SSRIs to be associated with long-term risk of weight gain.^79, 89, 91, 92^ However, the number of long-term studies over the period of 1 year is limited, and there is a need for studies specifically investigating the long-term effects of SSRIs on body weight.

Cascade *et al.* reported that 6% of patients using SSRIs gained weight.^79^ The Canadian National Population Health Survey (NPHS) is a longitudinal study that has found that SSRIs and the SNRI venlafaxine use are associated with significant weight gain.^89^ A long-term comparison study investigated the effects of different types of SSRI on weight gain in patients with panic disorder. It showed that paroxetine, fluoxetine, citalopram and fluvoxamine induced weight gain of, respectively, 8.2±5.4, 5.2±4.4, 6.9±5.7 and 6.3±4.2 kg in a 1-year period.^91^ Michelson *et al.* observed changes in body weight during a 1-year trial period of fluoxetine treatment. The initial 12-week period was associated with weight loss of 0.35 kg, and 50 weeks of treatment resulted in weight gain of 3 kg.^92^

In a prospective 4-year follow-up study, SSRI-treated patients had weight gain of 4.6%.^88^ In the study conducted by Sussman *et al.*, 17.9% of SSRI users experienced significant weight gain ⩾7%.^93^ Escitalopram (SSRI) treatment resulted in an increase of 0.05 in BMI scores during the first 12 weeks, and there was an increase of 0.12 in BMI scores after 6 months of treatment initiation.^94^

In addition to the clinical studies described above, case studies have reported extreme weight gain in patients with SSRI treatment.^97, 98^ In the study conducted by Bouwer *et al.*, treatment with citalopram led to weight gain and carbohydrate craving in eight out of eighteen patients. In a 4-week period, one female patient gained 8 kg, whereas in a 5-week period one male patient gained 9 kg.^98^ In another case study, a 33-year-old schizophrenic female patient was treated with fluoxetine (40 mg per day) for a 9-month period following antipsychotic treatment with risperidone (6 mg per day) and clorazepate (15 mg per day).^97^ The patient was discontinued from fluoxetine because she underwent excessive weight gain (52 kg) accompanied with carbohydrate craving.^97^ Therefore, some individuals may be more sensitive to weight gain induced by SSRIs than others.^97, 98^

## Meta-analysis study

Recently, Serretti *et al.* performed a meta-analysis on the effects of antidepressants on body weight.^95^ The study showed that acute treatment with fluoxetine and bupropion was associated with significant weight loss of 0.94 and 1.13 kg, respectively.^95^ Imipramine was associated with non-significant weight loss of 0.2 kg, and mirtazapine was associated with significant weight gain of 1.74 kg.^95^ When considering treatment periods longer than 4 months, imipramine was associated with non-significant weight loss of 0.04 kg, bupropion with significant weight loss of 1.87 kg and fluoxetine with non-significant weight loss of 0.31 kg.^95^ In contrast, mirtazapine was associated with non-significant weight gain of 2.59 kg (*P*<0.07) with upper limit of 5.41 kg.^95^

## Limitations of current findings

Although these previous studies described the effects of antidepressants on body weight, there are multiple problems in clinical observations because of the methods used. Most previous data on antidepressant-induced weight gain were extracted from drug trials of antidepressant medications or larger cross-sectional observational studies investigating the broad spectrum of MDD research. Confounding effects such as physicians specifically selecting antidepressant medication taking into consideration the risk of weight gain could not be eliminated.^89^ Moreover, as described previously, weight gain is one of the most undesired effects of treatment, and one of the major reasons for discontinuing treatment within the first 2 months of treatment initiation.^94^ In most cases there was no long-term follow-up reported that would allow us to ascertain the impact of antidepressant treatment or exposure over the lifetime.

## Mechanism proposed of antidepressant-induced weight gain

**Figure 1:**
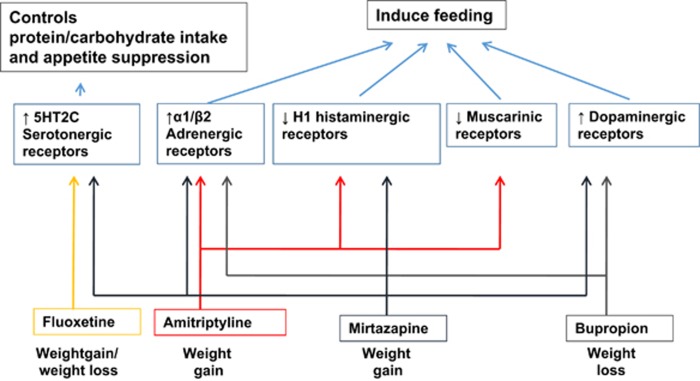
Mechanism of monoamine neurotransmission in antidepressant-induced weight gain. Note: ↑ denotes increased activation; ↓ denotes decreased activation.

Central and peripheral mechanisms of appetite and feeding behaviour regulation are complex; regulatory factors such as neurotransmitters, neuropeptides and hormone-like peptides have a role in orchestrating appetite, feeding behaviours and metabolic pathways.^99^ In previous studies, it has been suggested that weight gain associated with antidepressants reflects the action of monoamine pathways, which include serotonergic, adrenergic, histaminergic, cholinergic and dopaminergic receptors. The specific mechanisms of action of different antidepressants on weight are described below (Figure 1).

## Mirtazapine

Mirtazapine (SNRI) has been associated with weight gain. The mechanism of mirtazapine involves the blockade of α2-adrenergic receptors, together with affinity for histamine H1 receptors and low affinity for dopaminergic D1 and D2 receptors.^95^ It has been suggested that the histaminergic system is involved in appetite and feeding control.^100^ These histaminergic cell bodies are situated in a specific region called the tuberomammilary nucleus of the posterior hypothalamus.^100^ Histaminergic receptors have been shown to interact with orexigenic neuropeptides such as orexin A, NPY and ghrelin.^101^ Anti-histaminergic effects of mirtazapine were associated with enhanced appetite by activation of ghrelin and NPY.^95, 101^ Furthermore, clinical doses of mirtazapine blocked both 5HT2 and 5HT3 receptors.^99^ 5HT2 receptors have an essential role in appetite, and antagonising 5HT2 results in weight gain.^99^

## Bupropion

In contrast to mirtazapine, bupropion has been known to induce significant weight loss.^95^ The mechanism of action of bupropion is determined by its effect increasing dopamine and norepinephrine neurotransmission.^95^ Bupropion and its metabolites selectively inhibit dopamine and norepinephrine reuptake pumps, and may also alter the release of dopamine and norepinephrine; yet, it does not induce effects on postsynaptic receptors including histamine, α- or β-adrenergic, serotonin, dopamine receptors; it is also a weak nicotinic acetylcholine receptor antagonist. Moreover, reduced dopaminergic tone in the hypothalamus of obese individuals may be reversed by bupropion by increasing the activity of the hypothalamic melanocortin system, which is an important component in the regulation of homeostatic energy balance.^102^

## TCA (amitriptyline)

The pharmacological actions of TCAs are known to be non-selective and these results in side effects associated with significant weight gain. Amitriptyline has high affinity for α-adrenergic, histaminergic and cholinergic receptors.^95^

## SSRI (fluoxetine)

Fluoxetine, a SSRI, has weight-reducing effects acutely. Fluoxetine has significant affinity for 5HT2C receptors, which have a role in appetite suppression. In addition, fasting plasma leptin levels (an anorexigenic adipokine) were increased in patients treated with either imipramine or fluoxetine.^99, 103^ In an acute experiment, fluoxetine injections in obese and lean rats resulted in reduced food intake.^104^ In these rats, fluoxetine significantly reduced NPY levels in the paraventricular nucleus of the hypothalamus.^104^ Acute weight loss is due to regulation of the carbohydrate/protein ingestion ratio, which is known to occur through 5HT.^99^ Nevertheless, it would be essential to elucidate the mechanism of long-term effect of fluoxetine on body weight, which remains unclear.^99^

## Animal models of depression and obesity

### Animal models of stress and depression

In the past, different attempts have been made to develop an animal model that mimics the clinical manifestations of MDD.^105^ MDD is a complex disorder, and it remains controversial whether animal models of depression mimic the depressive state of humans. Therefore, Willner *et al.* proposed that animal models of depression should fulfil a set of validity criteria, which included predictive, face and construct validities.^106^ Predictive validity is based on whether the animal model shows reliable pharmacological effects of antidepressants.^107^ Face validity assesses both treatment features of chronic antidepressant therapy and symptomatic features.^107^ The meaning of construct validity is rather complex, and is defined as having both behaviour and features of depression such as 'helplessness or anhedonia'.^107^ Furthermore, arising evidence suggests that pathophysiological changes in the neuroendocrine system, such as in the HPA axis and hippocampal structural changes in the process of neurogenesis, may be important biomarkers to validate an animal model of depression.^105, 107^ According to the study conducted by Willner *et al.*, only a few models passed all three criteria, and these models include chronic unpredictable stress, learned helplessness and intracranial self-stimulation.^106^ For these reasons, most of these animal models are referred as 'animal models of stress' instead of 'animal models of depression'.^106^

In animal models, behavioural testing has been used to screen the antidepressant effects of drugs. The most classic tests are known as the forced swimming test and the tail suspension test. Both forced swimming test and the tail suspension test are based on the despair behaviour, identified with immobility and lack of motivation to escape, and antidepressants have been demonstrated to reverse these behaviours.^108, 109^ Behavioural tests that include novelty suppressed feeding, open field and elevated plus maze have been used to screen anxiety-related behaviours in animal models of depression,^110, 111^ whereas cognitive tests, such as the Morris water maze, have been used to assess spatial memories and cognitive deficits associated with hippocampal atrophy.^112^ Furthermore, anhedonic behaviour has been tested by the sucrose preference test.^113^

## Animal models of obesity

At present, there are various types of animal models of obesity. These include spontaneous mutants such as the ob/ob and the db/db mouse models, genetically engineered models of the *Mc4r* gene and those based on environmental factors such as diet-induced obesity (DIO).^114, 115, 116, 117^

## Animal models of genetic obesity

Leptin is an adipocyte-derived hormone encoded by the *ob* gene.^115, 116^ About two decades ago, the discovery that a genetic mutation in the *ob* gene caused leptin deficiency and severe obesity showed that leptin is a critical molecule in the regulation of energy expenditure, food intake and attenuation of adipose mass.^115, 116^ It was demonstrated that leptin has a high binding affinity in the hypothalamus, and it inhibited the orexigenic hormone NPY gene expression and synthesis.^118^

Further along, other obesity animal models have included the db/db model and melanocortin-4 receptor null mouse, which are characterised by elevated leptin levels and failure to attenuate obesity after leptin treatment, have provided the foundation for the concept of leptin resistance and hyperleptinaemia.^115, 117^

## Animal models of DIO

In 1949, animal models of DIO were initially achieved by *ad libitum* feeding of a semi-liquid palatable diet.^119^ Since then, there has been an extensive number of studies seeking to achieve an optimal method for DIO using various methods. These methods included determining the optimal composition and duration of a diet, age and the genetic background of an animal model to induce DIO (Table 2).^119^ Recently, a study conducted by Buettner *et al.* concluded that the optimal condition for DIO in an animal model involved a semi-purified high animal fat diet of 40% of energy, with a limited amount of 3-polyunsaturated fatty acids (3-PUFA), and plant oils rich in 6-PUFA and 9-PUFA.^119, 120^ Long-term DIO was accompanied with visceral obesity, glucose intolerance, hyperdyslipidaemia, hyperleptinaemia and hyperinsulinaemia.^121^

Optimal conditions for the DIO model involve genetically outbred environments as they provide genetic diversity, where some animals are prone to obesity while others are resistant to it.^119^ A well-established method for studying DIO in rodents involves classifying groups of animals as 'obesity-prone' and 'obesity-resistant' based on their body weight, body weight gain and/or body fat.^119^ Obesity-prone rats classified as early as after 5 days of high-fat-diet feeding already had distinguishable characteristics of metabolic dysregulation. Lipoprotein lipase activity, plasma leptin, triglycerides and glucose levels were all elevated.^125^ At week 5, obesity-prone rats had significantly higher epididymal and retroperitoneal fat pad weights in comparison with the obesity-resistant group.^122^ Metabolic changes included lipoprotein lipase activities, where the activities were higher in epididymal fat pads and lower in gastrocnemius muscles in the obesity-prone group.^122^ In C57BL/6 mice, mRNA levels of leptin receptor and NPY were elevated, whereas pro-opiomelanocortin mRNA expression was reduced in the obesity-prone group.^126^ Furthermore, plasma leptin and insulin levels were elevated, and there was increased activity of lipoprotein lipase in adipose tissue.^124^ In addition, there were increased glucose, non-esterified fatty acids, triglyceride levels in the plasma and elevated β-hydroxyacyl-CoAdehydrogenase activity in the muscle.^124^

## Animal models of stress and DIO

In animal studies, the linkage between stress and obesity remains non-consolidated, and the degree of stress paradigm, persistency or frequency of repeated stressors has a role in modulating body weight. Manting *et al.* demonstrated that, when a high-fat diet was given to rats, there was dysregulation in lipid metabolism.^127^ Moreover, when the high-fat diet was given in the presence of chronic stress, there was exacerbation in lipid dysregulation, where higher levels of triglycerides and total cholesterol, and lower levels of HDL-cholesterol were observed.^127^ In another study, the combination of social stress and a prolonged high-fat diet led to consequent dysregulation in lipid metabolism, enhanced plasma levels of non-HDLs and accumulation of triglycerides in the intrahepatic tissue.^128^ On the other hand, when a high-fat diet was given to the flinder-sensitive line rat genetic model of depression, depressive-like behaviour was exacerbated.^129^ These animal studies highlight the fact that on top of the of high-fat diet effect, stress can exacerbate metabolic dysregulation.

## Animal models of antidepressant and paradoxical weight loss

Currently, the existing animal models suggest that TCA and SSRI antidepressant administrations have been associated with 'paradoxical' weight loss and reduction in food consumption (Table 3). However, the relationship between antidepressant use, MDD and weight gain is complex; until recently, there had not been an animal paradigm that reproduced antidepressant-induced weight gain as observed in the clinical setting. In recent years, a novel animal paradigm that explains the weight trajectory of rodents during antidepressant treatment was developed by Mastronardi *et al.*^130^ This animal model involves stress-induced weight loss and consumption of a high-fat diet, aiming at following the long-term impact of antidepressant treatment in rodents. The novel animal paradigm suggests that short-term antidepressant treatment and stress, followed by a high-fat diet and long-term follow-up, has an important role in consolidating the role of antidepressant use in weight gain.

## Conclusion and future directions

In this review, we have discussed the relationship between MDD and obesity. Clinical findings have suggested that obesity could increase the risk of developing MDD, and *vice versa*. Several pathways may have a role in this interaction, including neuroendocrine, neuroimmune and neurotropic mechanisms. Among those, activation of the HPA axis occurs both during MDD/stress and obesity, making it the most accepted shared common pathophysiological pathways in both disorders. Leptin and insulin resistance are pathophysiological mechanisms that need to be further elucidated, along with the roles of the immune system and neurotropic factors.

Despite the concomitant occurrence of the frequent use of antidepressants and the high incidence of obesity in Western societies, additional studies are required to fully test our hypothesis that the rise in obesity rates is related at least in part to increasing antidepressant use, and to elucidate the mechanisms underlying antidepressant-induced weight gain. Numerous studies have investigated the effects of different classes of antidepressants on body weight. Previous clinical studies suggest that TCAs amitriptyline, nortriptyline and imipramine were associated with weight gain. Mirtazapine (SNRI) has been associated with increased weight gain. Studies have suggested that weight gain associated with antidepressants reflect the action of monoamine pathways, which include serotoninergic, adrenergic, histaminergic, cholinergic and dopaminergic receptors. It has been suggested that the mechanism behind weight gain induced by TCAs and mirtazapine (SNRI) involves affinity for histaminergic receptors. Histaminergic receptors have been shown to interact with orexigenic neuropeptides such as orexin A, NPY and ghrelin. Bupropion (SNRI) has been shown to induce significant weight loss via selective inhibition of dopamine. On the other hand, because of dispersion in clinical studies, the effect of SSRI on body weight remains still unclear. Despite the fact that SSRI use has been associated with weight loss during acute treatment, a number of studies have shown that SSRIs may be associated with long-term risk of weight gain. Long-term studies with very much extended periods are needed in order to understand long-term effects of SSRI on body weight.

In animal studies, the presence of stress in addition to high-fat diet has been shown to exacerbate metabolic dysregulation. However, further efforts to develop animal models to characterise and consolidate the linkage between stress and obesity are needed. Important factors that need to be considered in developing animal model of stress and obesity are the degree of stress paradigm, persistency or frequency of repeated stressors, type of diet used and duration of the study. At present, antidepressants have shown to induce 'paradoxical weight loss' and reduction in food consumption in most animal models; however, a newer paradigm shows that the combination of stress and antidepressants followed by long-term high-fat diet results in markedly increased weight, in excess of what is caused by high-fat diet alone.

In this review we highlight the fact that the relationship between antidepressant use, MDD and weight gain is complex. We emphasise the fact that it is important to develop animal paradigms that better mimic the clinical settings, and include lifestyle factors such as dietary choices. Furthermore, future studies are needed to elucidate the mechanisms underlying depression, obesity and antidepressant-induced weight gain, and to test the hypothesis proposed in this paper that the increasing exposure to antidepressants, defined as current or past use of antidepressant drugs, in the context of obesogenic diets and environments, is a contributory factor to the obesity epidemic.

## Figures and Tables

**Table 1:** Clinical studies on the effects of antidepressants on body weight

*Study*	*Significance*	*Sample size/duration*	*Limitation*
Major depression, antidepressant medication and the risk of obesity^89^	SSRI and venlafaxine were significantly associated with obesity.There was no significant association between TCA or antipsychotic medications with obesity	NHPS sample (1994–2004) *n*=17 276	Confounded by prescription. Physicians may have specifically selected these medications for use in patients who they believe to be most at risk of weight gain
			
MetS abnormalities are associated with severity of anxiety and depression and with tricyclic antidepressant use^87^	Tricyclic antidepressants increased the odds for MetS	*n*=2981	The main focus of this study is on MetS and different levels of depression
			
Long-term weight gain in patients treated with open-label olanzapine in combination with fluoxetine for major depressive disorder^90^	Patients were treated with a combination of olanzapine and fluoxetine (OFC). Increases in fluoxetine dose were predictors of weight gain. Long-term (76 weeks) OFC treatment may lead to a large percentage (56%) of patient meeting the criteria for significant weight gain (>7%)	*n*=549	
			
Real-world data on SSRI antidepressant side effects^79^	36% of patients experienced side effects associated with SSRI. Forty-nine patients had weight gain	*n*=700 Patients	
			
A naturalistic long-term comparison study of selective serotonin reuptake inhibitors in the treatment of panic disorder^91^	Weight gain Paroxetine: 8.2±5.4 kg Fluoxetine: 5.2±4.4 kg Citalopram: 6.9±5.7 kg Fluvoxamine: 6.3±4.2 kg	Duration: 1 year *n*=200	
			
Changes in weight during a 1-year trial of fluoxetine^92^	12-Week treatment: –0.35 kg 50-Week treatment: +3 kg	Duration: 50 weeks *n*=395	
			
Changes in body weight during treatment with the new antidepressant Nefazodone, three SSRIs Fluoxetine, Setraline, Paroxetine, and the tricyclic Imipramine^93^	Significant weight loss or gain was, respectively, defined as ⩽7 and ⩾7% change in body weight from baseline. Study 1 Acute phase trial SSRI: 4.3% of treated patients lost weight at any point Nefazodone: 1.7% of treated patients lost weight at any point Long-term phase trial SSRI: 17.9% of treated patients had weight gain Nefazodone: 8.3% of treated patients had weight gain Study 2 Acute phase trial Imipramine: 4.9% of treated patients had weight gain Nefazodone: 0.9% of treated had weight gain Long-term phase trial Imipramine: 24.5% of treated patients had weight gain Nefazodone: 9.5% of treated patients had weight gain	Study 1 Acute phase trial: 6–8 weeks *n*=1036 Long-term phase trial: 16–46 weeks *n*=608 Study 2 Acute phase trial: 6–8 weeks *n*=1036 Long-term phase trial: 16–46 weeks *n*=135	
			
TCA-induced weight gain^76^	TCA antidepressants Amitriptyline, nortriptyline and imipramine induced weight gain of 1.3–2.9 lbs per month, and weight increased linearly overtime	*n*=40 Average of 6-months treatment	
			
Body weight gain during nortriptyline (TCA) or escitalopram (SSRI) treatment^94^	Nortriptyline First 12 weeks: +1.22 kg, BMI score increase of 0.44 After 6 months: +1.82 kg, BMI score increase of 0.64 Escitalopram First 12 weeks: +0.14 kg, BMI score increase of 0.05 6 Months: +0.34 kg, BMI score increase of 0.12	*n*=630 12 weeks and 6 months of treatment	
			
Weight gain associated with tricyclic or SSRI treatment^88^	Average weight gain of 1.4 kg (2.5%) in the control group and 2.5 kg (4.3%) among users of 200 defined daily doses of antidepressant	*n*=5537 4 years	

Abbreviations: BMI, body mass index; MetS, metabolic syndrome; NHPS, National Population Health Survey; OFC, a combination of olanzapine and fluoxetine; SSRI, selective serotonin reuptake inhibitor; TCA, tricyclics.

**Table 2:** Animal models of diet-induced obesity

*Study*	*High-fat diet*	*Metabolic factors*	*Study duration*
Pagliassotti *et al.*^122^	60% of kcal from fat	Plasma level of insulin, β-hydroxybutrate, mRNA level and activity of lipoprotein lipase were higher in epididymal fat pad and lower in gastrocnemius muscle	5 Weeks
			
Huang *et al.*^123^	59% of kcal from fat	Increased mRNA levels of leptin receptor and neuropeptide Y. Decreased POMC mRNA level	13 Weeks
			
Dourmashikin *et al.*^124^	50% fat content	Increased levels of leptin, insulin and lipoprotein lipase in adipose tissue. Hyperphagia, enhanced circulating triglycerides, nonesterified fatty acids and enhanced glucose, galanin. Increased β-hyroxyacyl-coaldehydrogenase level in muscle	3 Weeks
			
Dourmashikin *et al.*^125^	45–60% fat content	Elevated leptin, insulin, triglyceride, glucose, lipoprotein lipase activity in adipose tissue	4–6 Weeks
			
Madsen *et al.*^121^	31.8% fat content	Development of visceral obesity, hyperleptinaemia, hyperinsulinemia and dyslipidaemia, and decrease in glucose tolerance	9 Months

Abbreviation: POMC, pro-opiomelanocortin.

**Table 3:** Effects of antidepress ants on the body weight of animal models

*Species*	*Treatment/duration*	*Proposed mechanism/pathway*	*Effect on body weight*	*Diet*	*Reference*
Fa/Fa Zuker rats	Fluvoxamine (25 mg kg^−1^) treatment for 7 days	SSRI induces weight loss by increasing CRH level at the paraventricular nucleus of hypothalamus	Weight loss	Chow diet	Wieczorek *et al.*^131^
					
Wild-type Ay mice	Treatment of 30–60 mg kg^−1^ of milnacipran followed by food presentation after 30 min. Food intake measured after 1/6 h. Study duration: 3 days	Increased hypothalamic POMC and CART	Decreased food intake and weight	Chow diet	Nanogaki *et al.*^132^
					
Mice	Fluoxetine treatment/48 h of fasting		Decrease in body weight and food intake with fluoxetine treatment	Chow diet	Sullivan *et al.*^133^
					
Rats	Early-life neonatal handling stress followed by 0.25 mg kg^−1^ per day of imipramine treatment from days 60 to 120		Chronic imipramine treatment decreased craving for sweet pellets	Chow diet	Portella *et al.*^134^
					
Female rats	30 days of chronic stress (food deprivation, restraint, forced swimming test and flashing light stress) followed by 60 days of fluoxetine treatment		Decreased consumption of sweet pellets with fluoxetine treatment	Chow diet	Gamaro *et al.*^135^
					
Rats	7-day treatment of fluoxetine or imipramine 10 mg kg^−1^ with repeated restraint stress, followed by 177 days of high-fat diet during post-stress period		Increased body weight and absolute caloric intake during post-stress period		Mastronardi *et al.*^130^

Abbreviations: CART, cocaine and amphetamine-regulated transcript; CRH, corticotropin-releasing hormone; POMC, pro-opiomelanocortin.
